# Cost analysis of centralized viral load testing for antiretroviral therapy monitoring in Nicaragua, a low-HIV prevalence, low-resource setting

**DOI:** 10.1186/1758-2652-13-43

**Published:** 2010-11-05

**Authors:** Jay Gerlach, Magda Sequeira, Vivian Alvarado, Christian Cerpas, Angel Balmaseda, Alcides Gonzalez, Tala de los Santos, Carol E Levin, Juan Jose Amador, Gonzalo J Domingo

**Affiliations:** 1 Programs for Appropriate Technologies in Health (PATH), Seattle, Washington 98121, USA; 2 PATH, Managua, Nicaragua; 3 Centro Nacional de Diagnóstico y Referencia, Costado Oeste Colonia Primero de Mayo, Managua, Nicaragua

## Abstract

**Background:**

HIV viral load testing as a component of antiretroviral therapy monitoring is costly. Understanding the full costs and the major sources of inefficiency associated with viral load testing is critical for optimizing the systems and technologies that support the testing process. The objective of our study was to estimate the costs associated with viral load testing performed for antiretroviral therapy monitoring to both patients and the public healthcare system in a low-HIV prevalence, low-resource country.

**Methods:**

A detailed cost analysis was performed to understand the costs involved in each step of performing a viral load test in Nicaragua, from initial specimen collection to communication of the test results to each patient's healthcare provider. Data were compiled and cross referenced from multiple information sources: laboratory records, regional surveillance centre records, and scheduled interviews with the key healthcare providers responsible for HIV patient care in five regions of the country.

**Results:**

The total average cost of performing a viral load test in Nicaragua varied by region, ranging from US$99.01 to US$124.58, the majority of which was at the laboratory level: $88.73 to $97.15 per specimen, depending on batch size. The average cost to clinics at which specimens were collected ranged from $3.31 to $20.92, depending on the region. The average cost per patient for transportation, food, lodging and lost income ranged from $3.70 to $14.93.

**Conclusions:**

The quantitative viral load test remains the single most expensive component of the process. For the patient, the distance of his or her residence from the specimen collection site is a large determinant of cost. Importantly, the efficiency of results reporting has a large impact on the cost per result delivered to the clinician and utility of the result for patient monitoring. Detailed cost analysis can identify opportunities for removing barriers to effective antiretroviral therapy monitoring programmes in limited-resource countries with low HIV prevalence.

## Background

Recent progress in managing antiretroviral therapy (ART) in patients with HIV/AIDS in low-resource settings has outpaced the scaling-up of tests to support effective case management. CD4 and HIV viral load are the two primary tests used to monitor viral suppression.

Cost-effectiveness studies are fairly concordant on the added value of CD4 testing versus symptom-based management of patients on ART [1-4]. Less clear is the cost effectiveness of viral load testing for management of patients on ART [2,3,5,6]. Compared with CD4 testing, viral load testing is more expensive and technically complex. Yet there is increasing awareness of the benefit of viral load testing both for patient management and for public health in terms of early and accurate detection of failure to respond to ART, thus mitigating drug resistance and averting new infections [7-10].

To date, detailed cost analyses of viral load testing have focused primarily on the activities that take place after a specimen arrives at a viral load testing facility to the point at which the test results are generated [11-15]. The studies did not take into account patient costs, the costs of specimen collection and transport, or added costs resulting from inefficiencies in any aspect of the process, both from the patient perspective and from the specimen collection site (SCS) perspective.

In addition, most of these reports evaluating ART strategies in low-resource settings have focused primarily on sub-Saharan Africa, where the AIDS burden is highest. These reports have ignored the challenges in implementing a sustainable ART monitoring programme in low-prevalence countries, which are likely to be different from those in high-prevalence countries.

To increase understanding of the costs of viral load testing to patients and health systems in low-HIV prevalence settings, we performed a cost analysis in Nicaragua, a country with an adult HIV prevalence of 0.2% (range: 0.2% to 0.4%) [16]. Our objectives were to assess total costs, identify inefficiencies in the systems supporting viral load testing, and identify current and emerging solutions that could make viral load testing more accessible in low-prevalence countries.

## Methods

### Study sites

In collaboration with the Nicaraguan Ministry of Health, the research team selected six SCS for data collection. The six SCS facilities are distributed over five regions of Nicaragua: Managua; a Pacific coast region; a central region; and two Atlantic coast regions. With the exception of one health centre site, all are regional referral hospitals. Data were also collected from the Centro Nacional de Diagnóstico y Referencia (CNDR) in Managua, Nicaragua's national reference laboratory, where all viral load testing is performed.

### Data collection

From June 2008 until June 2009, data were compiled and cross referenced from multiple information sources: laboratory records, regional surveillance centre records, and scheduled interviews with the key healthcare providers responsible for HIV patient care. Patient costs were obtained indirectly from multiple sources, including municipal sources, Ministry of Transportation records, hostelling sites, and non-governmental organizations working with HIV patients. We used direct observation to estimate the resources used for viral load testing, and accessed CNDR administrative records to obtain 2008 purchase costs for testing supplies.

Input, resource use and unit cost data were collected for the following steps and activities:

1. Patients travelling to collection sites and providing a sample for viral load and CD4 testing.

2. Health workers collecting the specimen at a specimen collection site (SCS) to obtain a 10 ml blood sample for the viral load test.

3. Health workers at the SCS shipping the specimen to the testing facility.

4. Laboratory workers performing the viral load testing.

5. Testing facility staff communicating test results to the ART managing facility. These data were not collected directly, but were included in the administrative costs at the CNDR, Sistemas Locales de Atención Integral en Salud (SILAIS, Local System of Integrated Health Care), the SCS and the ART management facility.

### Cost categories

For our analysis, we estimated the cost of viral load testing that accrued to three parties: the patient; the clinic or hospital at which the specimen was collected; and the laboratory at which the test was performed.

### Patient costs

We estimated patient costs associated with transportation to and from the SCS, the purchase of food that otherwise would not have been purchased as part of the daily routine, overnight accommodations (if necessary), and lost income as a result of taking the time to have the specimen collected. To evaluate the full range of patient experiences, these costs were calculated for patients from all regions served by the respective SCS facilities.

To facilitate comparison between regions and to calculate the average patient cost nationally, we estimated weighted average patient costs based on the place of residence. The weighted average patient cost was calculated by multiplying the average cost per patient from each location by the number of patients from each location. Thus, if 80% of patients lived near the SCS and only 20% were remote, the weighted average cost would be closest to the cost for those who lived near the SCS.

### Clinic/hospital costs (SCS)

SCS costs included labour associated with all required staffing levels, specimen collection supplies, specimen transport and storage supplies, and annualized depreciation costs for capital equipment. Additional costs included accommodations and/or food when the clinic provided these to the patient.

Supplies included the full range of materials used to collect and transport the specimen, including latex gloves, vacutainers, waste disposal boxes, and thermoses for cold transport. Labour was broken into the following categories: registration; administration (including results processing); site preparation; patient counselling; and specimen collection, processing and packaging. Transport costs for shipping the specimen to the testing facility included labour for a driver, fuel consumption, vehicle depreciation and airfare (if necessary). Since HIV prevalence is low in Nicaragua, the overhead costs attributable to ART management are low in comparison with all other hospital costs. Overhead facility costs therefore were not represented in the analysis.

### Laboratory costs

The team collected information on inputs for resources and unit costs associated with specimen processing and viral load testing, such as laboratory supplies and equipment and waste management. At the national lab, the CNDR performed viral load testing with the Roche Molecular Diagnostics COBAS^® ^Amplicor test kit until the end of 2009. The 2008 price to the CNDR for the purchase of COBAS^® ^Amplicor reagents was used in this cost analysis. To determine labour and materials at this level, we conducted a time and motion study of laboratory procedures for viral load testing. Laboratory costs were classified as labour, annualized depreciation for capital equipment, disposable supplies (including test kits), and facility overhead costs.

In addition, we captured information on mean time for results to be delivered from the CNDR reference laboratory to the SCS, the percentage of samples collected at the SCS for which results were returned to the SCS from the CNDR, and estimates on loss to follow up. Data for the results returned to the SCS were collected from laboratory and SCS records. Specifically, we collected data to calculate the number of runs performed per month, the number of tests performed per month, the average number of tests per run, the maximum delay in performing tests, the level of batching, and the labour time to perform a viral load test (including the sub-steps). Anecdotal estimates on loss to follow up were obtained from the ART multidisciplinary team at the SCS.

The costs associated with communicating test results back to the ART managing facility were included in the administrative costs of the CNDR, the SILAIS and the ART management facility.

### Data analysis

We developed a Microsoft Excel-based cost model to estimate: (1) the per-patient cost per specimen collected; (2) the per-SCS cost per specimen collected; and (3) the cost per test performed at the CNDR laboratory.

In addition, we developed a module that took into account the number of tests in each kit configuration and the laboratory's specific throughput (number of tests run). This allowed us to vary the laboratory costs depending on batch size. The CNDR laboratory typically uses optimal batch sizes for viral load testing. Using specific data from the laboratory on its optimal batch size (i.e., the number of specimens per batch), we estimated the laboratory cost per test performed based on varying batch sizes, from one to 24 specimens. An optimal batch size is 22 specimens. Running a batch of 10 specimens is more cost effective than running batches with 11 to 19 specimens. Ten and 22 specimen batches are possible when including only two controls (one negative and one positive) per run. Thus, for the CNDR laboratory, we estimated a typical batch run as either 10 or 22 specimens, depending on demand for testing.

After calculating the cost per specimen collected and viral load test performed, we calculated: (1) the cost per result delivered to the SCS; and (2) the total cost per result delivered to inform patient care.

At the SCS level, we calculated the cost per viral load test result communicated back to the SCS, using rates of attrition for specimens sent to the CNDR and results returned to the SCS as obtained from laboratory and SCS records. To calculate the cost per viral load test result communicated back to the patient, we used rates for loss to follow up collected from records or anecdotally at the SCS.

### Human subjects and research determination

This study was reviewed by the Programs for Appropriate Technologies in Health (PATH) Research Determination Committee. Informed consent was not required, as the data were collected from public records and analyzed anonymously. Patients were not interviewed or contacted for data collection, and no human identifiers were collected.

## Results

### Viral load testing in Nicaragua

In May 2008, ART distribution in Nicaragua was decentralized to 16 hospitals and three health centres throughout the country. As of November 2008, 78% of the SILAIS facilities provided ART. National regulations provide for viral load and CD4 testing every six months for each HIV patient. At the time of this study, the CNDR in Managua was the only laboratory performing viral load measurements and CD4 determinations. Thus, all specimens were sent to the CNDR laboratories in Managua for CD4 and viral load testing.

**Figure 1:**
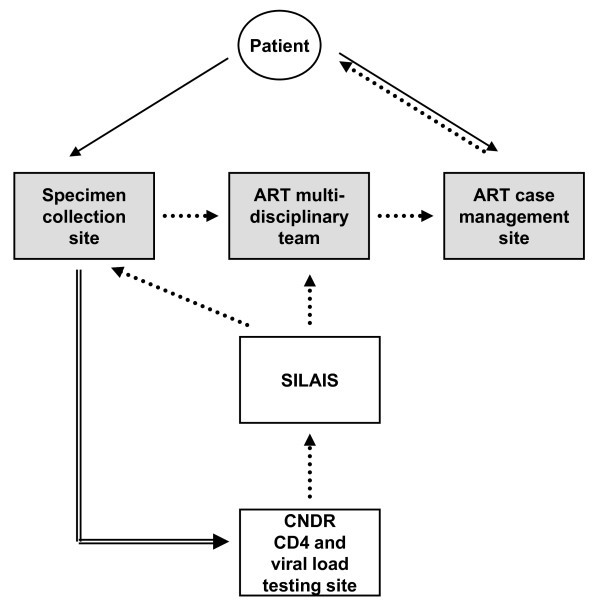
**Summary of process for CD4 and viral load testing**. The patient goes to the SCS (solid arrow) to have blood drawn. The specimen is shipped under the cold chain to the central laboratory (CNDR) for CD4 and viral load testing (double-line arrow). Results are communicated (dotted arrow) back to the originating local healthcare department (SILAIS). The SILAIS then communicates the results back to the pertinent ART multidisciplinary team. The SCS, ART multidisciplinary team and ART case management site (gray shading) may or may not reside at the same healthcare facility.

Specimens were collected at SCS facilities for both CD4 and viral load testing. Three EDTA-K_3 _BD vacutainers^® ^of blood were collected: one for CD4 and two for viral load. The vacutainers were sent to the CNDR laboratories on ice and had to arrive within six hours of collection time to be considered valid. CD4 tests were performed on the date of arrival at the CNDR. Blood collection tubes for viral load were centrifuged upon arrival to the CNDR and the plasma from the samples stored at -70°C. Plasma samples were allowed to accumulate until they could be batched in the viral load assay for optimal throughput testing. The CD4 and viral load test results were independently entered into a report that was communicated to the originating SILAIS, which then communicated the results to the ART multidisciplinary team. ART management may or may not have occurred at the same site as specimen collection (Figure 1).

For this study, detailed cost data were collected from six nationwide SCS facilities and the CNDR laboratory. The SCS facilities represent urban, rural and remote settings, with four sites representing regions with urbanization levels of 30% or less. Each site submitted between 30 and 330 specimens for viral load testing in 2008.

### Total cost per test

**Figure 2:**
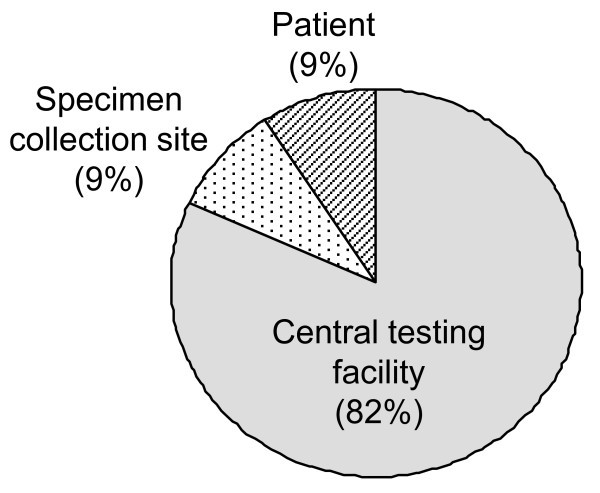
**Relative patient, SCS and central testing facility costs to perform an HIV viral load test**.

Table 1 shows a summary of the costs per test performed to patients, the SCS and the viral load testing laboratory. The costs ranged from $99.01 to $124.58. In all cases, laboratory costs represented the greatest share of the total cost. There was some variability in the percentage of the total cost per patient versus the percentage of the total cost per SCS across regions. On average, however, the overall cost per patient was similar to the overall cost per SCS (Figure 2).

**Table 1:** Total patient, SCS and laboratory costs per viral load test performed, by site (US dollars)

SCS site	1	2	3	4	5	6
**Patient**	3.70 (3.7%)	8.90 (8.8%)	8.78 (8.5%)	10.27 (9%)	14.93 (12%)	14.62 (13%)
**Clinic**	6.58 (6.7%)	3.31 (3.3%)	6.41 (6.1%)	14.97 (13.1%)	20.92 (16.8%)	9.48 (8.4%)
Laboratory*	88.73(88.6%)	88.73(87.9%)	88.73 (85.4%)	88.73 (77.9%)	88.73 (71.2%)	88.73 (78.6%)

**Total**	**99.01**	**100.94**	**103.92**	**113.97**	**124.58**	**112.83**

*Calculated using the optimal 22 specimens perrun.

### Patient costs

**Figure 3:**
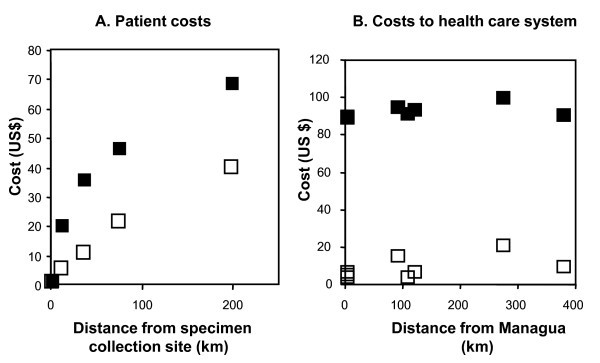
**Relationship between costs and distance**. 3A. The distance from home to the SCS for HIV patients in the Atlantic regions. Empty squares represent the direct transportation costs incurred by patients. Filled squares represent fully burdened food, lodging and opportunity costs to patients. 3B. The distance from the SCS to the central testing facility (in Managua). Empty squares represent specimen transport costs. Filled squares represent the cost per viral load test to the healthcare system.

Minimum and maximum costs indicate that proximity to the SCS is the main driver of cost to patients (Table 2), with transportation being the main cost contributor. Larger urban sites with a small minority of patients living in remote areas had lower weighted average patient costs (e.g., Site 1). In contrast, in more rural regions, where a larger proportion of patients live in remote areas, the weighted average patient cost was as high as $14.93. The majority of patients - that is, those who live near the clinic - bear a cost well below the average patient cost, whereas the minority of patients who live in remote areas bear a cost well above the average. Overall, 65% of the patients bear a cost of less than $5 for providing a sample, and 95% bear a cost of less than $10. In the Atlantic regions, the terrain imposes significant access challenges, requiring patients to spend up to three days to complete the process of providing a specimen. The relationship between patient distance from the SCS at one Atlantic coast site and cost to the patient is shown in Figure 3A.

**Table 2:** Patient costs, by site (US dollars)

SCS site	1	2	3	4	5	6
Transportation*	1.23 (0.42/4.50)	1.54 (0.42/3.83)	2.13 (0.42/3.83)	2.85 (0.37/7.73)	6.64 (1.05/40.05)	5.68 (1.05/105.20)
Lodging*	0.1 (0/0.10)	1.74 (0/4.34)	1.16 (0/6.31)	0 (0/0)	2.28 (0/6.48)	1.78 (0/23.67)
Food*	0.86 (0.81/1.10)	2.26 (1.05/3.42)	3.37 (1.58/5.52)	2.17 (1.05/3.42)	3.37 (0/11.67)	4.63 (2.63/26.83)
Lost income*	1.40 (0.91/3.42)	3.36 (1.31/5.25)	2.12(1.06/4.25)	2.39 (0.42/6.66)	2.64 (0.0/10.82)	2.54 (1.15/18.44)

**Total weighted average**	3.70	8.90	8.78	10.27	14.93	14.62

**Total median**	2.14	5.88	7.71	8.13	1.69	4.83

**Min/max**	2.14/8.97	2.78/16.85	3.07/19.92	2.20/25.54	1.05/69.03	4.83/174.14

*Minimum and maximum costs are provided in parentheses.

### Specimen collection site costs

Our analysis shows that the cost per test performed at the SCS ranged from $3.31 to $20.92 (Table 3). The largest contributors to cost differences for the SCS were transport and labour, although there were significant differences in costs for supplies and patient accommodations/food. Sites 5 and 6 experienced the highest transportation costs because they had to dispatch the specimens to the CNDR by air. Batching specimen collection and shipment achieved a lower cost per specimen (Sites 5 and 6). A direct relationship between distance of the SCS and the central laboratory and the costs was not observed (Figure 3B).

**Table 3:** SCS costs, by site (US dollars)

SCS site	1	2	3	4	5	6
Supplies	1.80	0.56	0.59	0.90	0.61	0.50
Labour	4.66	0.66	1.66	5.66	6.66	7.66
Patient accommodations	0	0	0	2.77	2.89	0
Transport	0.13	2.10	4.16	5.64	10.76	1.33

**Total**	**6.58**	**3.31**	**6.41**	**14.97**	**20.92**	**9.48**

### Viral load testing laboratory costs

The CNDR used the Roche COBAS^® ^Amplicor test kit until 2009. The test kit's packaging and performance are configured to create economies of scale (data not shown). The optimal batch sizes and those performed by the laboratory are 10 or 22 specimen batches. Table 4 shows the per-specimen costs for batches of these sizes. The cost of the COBAS^® ^Amplicor test kit accounted for 90% of the cost of performing each test.

**Table 4:** CNDR laboratory costs for running viral load tests (US dollars)

	Batch of 10 specimens	Batch of 22 specimens
**Reagents (Amplicor test kit)**	**87.50**	**79.55**
		
**Disposables**		
Specimen aliquoting	0.19	0.17
RNA extraction	1.39	1.22
PCR reaction preparation	1.27	1.16

**Total disposables**	**2.85**	**2.54**

		
**Labour**		
Specimen receipt and processing	0.06	0.04
RNA extraction	0.23	0.19
PCR reaction preparation	0.12	0.06
Results processing	0.11	0.07
Administration	0.47	0.47
Data entry	0.20	0.20

**Total labour**	**1.03**	**1.19**

		
**Facilities and equipment**	**5.62**	**5.62**
		

**Total cost per specimen**	**97.15**	**88.73**

Facilities and equipment were the next largest contributors to cost, accounting for approximately 6%. Labour and disposables accounted for roughly 4% of the cost of performing the test.

### Cost of delivering test results

Table 5 shows the cost of delivering the viral load test results to the SCS and the patient, based on specific estimates of process efficiency and loss to follow up. In contrast with the costs to patients, the SCS and the testing laboratory (which ranged from $99.01 to $124.58, including administrative costs), the total cost per viral load test result returned to the SCS ranged from $100.94 to $153.80. These figures were based on total attrition rates obtained from the number of specimens sent to the central laboratory and the number of results returned to the SCS. It thus included both rejected samples and inefficiencies in results reporting.

**Table 5:** Total health system cost per test results delivered to the SCS and to the patient

SCS	Minimum	Mean	Median	Maximum
**% of results successfully delivered to the SCS**	81%	93%	92.5%	100%
**Total cost per results delivered to SCS (US$)**	100.94	118.94	114.08	153.80
**% of results delivered to the patient**	80%	88%	90%	99%
**Total cost per results delivered to patient (US$)**	112.15	134.63	135.48	155.35

**Increase in cost due to attrition in results delivered**	11%	23%	24%	33%

We recorded the time lapse for result delivery based on specimen shipment dates and result receipt dates at the SCS. The mean time for test result delivery per region ranged from 15 to 60 days and was not related to distance of the SCS from the CNDR laboratory in Managua. The time delay in test result delivery impacted the use of the results in informing treatment decisions, as well as actual delivery of the results to the patient. The total cost per result delivered to the patient was based on records and anecdotal data. Attrition in the results delivery process increased the cost per delivered test result by 11% to 33%, depending on the site (Table 5).

## Discussion

The aim of this study was to obtain a detailed cost profile for the process of HIV viral load testing in Nicaragua in order to identify inefficiencies in the systems that support viral load testing. With this information, we sought to identify opportunities to maximize access to viral load testing and generate evidence on the role of new and appropriate technologies.

### Cost implications for patients

The patient's cost burden for providing a specimen for ART monitoring is significant. On average, it is equivalent to the cost per specimen at the SCS (Figure 2). Patient cost is also subject to the greatest variability. In this study, we considered only travel costs, lodging and opportunity costs (as defined by days of work lost) as a result of providing a specimen. Based on these factors, the patient's distance from the SCS had the biggest impact on the cost of specimen collection for viral load testing, more than the distance of the SCS to the CNDR in Managua (Figure 3). The average monthly incomes in the regions where the study was performed were between $90 and $117 per month at the time of the study, so average patient costs of $3.70 to $14.93 is a significant burden to the patient.

For patients in remote regions, ART management has been decentralized to local healthcare providers, which means that patients do not need to travel to the SCS for ART. While this study did not examine adherence and reasons for failing to adhere to ART, other studies have shown that travel from the patient's home to the ART clinic is a major barrier [17-19]. In a similar way, our data suggest that programmes seeking to increase access to ART monitoring tests must consider patient costs, which can present a major barrier to testing compliance.

### Cost of testing kits

The viral load test itself remains the single most expensive component of the whole testing process, representing approximately 80% of the total costs. The CNDR in Nicaragua used the Roche COBAS^® ^Amplicor test kit during 2009. Given the limited viral load testing volume, there is no justification for housing multiple platforms in Nicaragua. Nicaragua does not have access to Clinton Foundation-negotiated prices, but even at a reagent cost of approximately $20 per specimen, the reagents would remain the most expensive part of the process. Procurement arrangements most significantly impact the absolute cost of viral load testing, as has also been discussed to be the case for CD4 testing [20]. Additionally, as demonstrated previously in a detailed cost analysis for viral load testing, the number of specimens tested per run can be the most impactful variable on the cost per reportable tests result [11,13].

Cost-saving opportunities could result from the adoption of lower-cost real-time polymerase chain reaction (PCR) tests or the Cavidi ExaVir^® ^reverse transcriptase assay [14,21-23]. We calculated, as described for the Roche test, the cost of performing the ANRS homebrew real-time PCR test [14,21] in Nicaragua to be $21.92 per specimen, compared with $88.73 for performing the commercial test under current prices (unpublished data). It should be noted, however, that the challenges of multiple vendor sourcing for different test components and additional import costs may lower the cost benefit of such a solution [24].

### Test results delivery

Delivery of the test results back to the patient's ART healthcare provider is a critical component of the full testing process. The efficiency of this process is in part determined by the percentage of results that are successfully delivered to the healthcare provider. A higher-efficiency communication of test results would reduce the cost per successfully delivered test result. In addition, faster delivery of test results may increase the healthcare provider's active use of the results and inform ART management.

Due to the high costs involved in performing the viral load test, any attrition in the test result delivery process has a large impact on the total cost per reported result. Interestingly, we found that this process had a much higher attrition rate than specimen shipment under the cold chain. As shown in Table 5, although the efficiency of the process from the CNDR and SCS laboratory records is high (> 80%), this has a large impact on the overall cost per reported test result. Further attrition is observed when taking into consideration the patient's need to return for the results, although the anecdotal nature of these reports means that there is less certainty in these numbers.

A related issue is the time lapse between performance of the viral load test and delivery of the results to the patient's ART healthcare provider. To further investigate this delay, we mapped the process for returning the viral load test results to the healthcare provider. There is significant variation in the process, and depending on the specimen's region of origin, a minimum of five or seven information transfers is involved. In contrast with specimen shipment from the SCS to the CNDR, where the SCS laboratory is responsible for successful delivery of the specimen, the result reporting system has several intermediaries, possibly explaining the attrition rates and delays in result reporting.

The long lapse between specimen collection and test result delivery may limit the role of the test results in actively informing ART management. Quantifying this impact may be critical for evaluating the effectiveness of viral load testing in ART monitoring strategies.

### Limitations due to cold chain requirements

The cold chain requirements for specimen preservation (and the maximum time lapse of six hours from specimen collection to arrival at the CNDR testing facility) mean that specimen collection cannot be decentralized, with the biggest cost impact falling on the patient. Such technologies as the use of dry filter spots (i.e., dried blood spots) could overcome this challenge. Alternately, a robust low- to medium-throughput platform that could be run at the SCS would meet the needs of most patients (95%, at a per-patient cost of less than $10). Such technologies are beginning to emerge [23,25].

### Waste management

The study results allowed us to project some implications of decentralization of viral load testing on waste management. In the centralized testing system, the majority of waste, which includes specimen vacutainers and disposables and chemicals for performing the test, is contained at the CNDR laboratory. Other researchers have shown that the cost of waste disposal for viral load testing is less than 0.1% of the total cost, or $0.01 to $0.07 per test [11]. Although this cost is minimal, it is important to note that any decentralization of viral load testing will significantly increase the biohazardous, chemical and nonhazardous waste at decentralized facilities, which in turn could require waste management capacity building.

### Study limitations

The methods used to collect data in this study present several limitations. Most critically, patients were not approached for this study, so data regarding patient costs are based on anecdotal scenarios, local transportation and accommodation costs, and average local earnings. Also, the study did not collect specific cost and time information regarding communication of results to the patient. Furthermore, the process was mapped only as far as communication of the viral load test results to the healthcare provider managing the patient. Although data were collected by the same researchers at all sites, the results reporting data were collected by informal interview and from laboratory records, which may have underestimated the administrative costs of the testing process. Also, staff training costs and waste management costs were not included in the study.

## Conclusions

Nicaragua has implemented an efficient viral load and CD4 testing system for patients on ART. The largest variables in the overall viral load testing cost were the patient's proximity to the SCS and the cost of viral load testing itself.

Patients living in remote settings face the highest testing costs. Any efforts to increase access to viral load testing must take into consideration patient transportation and opportunity costs as potential barriers. The process by which results are communicated to the healthcare provider managing the ART regimen is especially important, as the efficiency of this process has a large impact on the total cost per successfully delivered test result. Additionally, because the time between result generation and result communication affects how the results are used, decreasing this time would be beneficial.

With regard to viral load testing, if optimal specimen batching is routinely performed at the central laboratories, the single most expensive component of the process is the price of the commercial viral load test to the programme. Additionally, a low-throughput device of 10 to 12 specimens per run (appropriate for use at the SCS), in combination with specimen stabilization technologies, such as one using dried blood spots [26], could overcome some inefficiencies in the current system.

Increasingly, there is recognition that no single health system change or technology solution will meet the diverse needs related to HIV viral load testing [24,27-29]. Detailed operational research is required to unravel current inefficiencies and barriers in ART monitoring and to define the portfolio of technologies and system strengthening opportunities that will address these issues.

## List of abbreviations

ART: antiretroviral therapy; CNDR: Centro Nacional de Diagnóstico y Referencia; PATH: Programs for Appropriate Technologies in Health; PCR: polymerase chain reaction; NIBIB: National Institute of Biomedical Imaging and Bioengineering; SCS: specimen collection site; SILAIS: Sistemas Locales de Atención Integral en Salud (Local System of Integrated Health Care); USAID: United States Agency for International Development.

## Competing interests

The authors declare that they have no competing interests.

## Authors' contributions

All authors have read and approved the final manuscript. JG developed the analysis tools; MS and VA collected all the data and contributed to the analysis; CC and AB contributed to the laboratory cost analysis; CL and GJD participated in the design of the study and analysis of the data; and TS, AG, JJA and GJD participated in the conception and design of the study and helped draft the manuscript.
